# Intraventricular Flow Simulations in Singular Right Ventricles Reveal Deteriorated Washout and Low Vortex Formation

**DOI:** 10.1007/s13239-021-00598-9

**Published:** 2021-11-30

**Authors:** Anna Grünwald, Jana Korte, Nadja Wilmanns, Christian Winkler, Katharina Linden, Ulrike Herberg, Sascha Groß-Hardt, Ulrich Steinseifer, Michael Neidlin

**Affiliations:** 1 Department of Cardiovascular Engineering, Institute of Applied Medical Engineering, Medical Faculty, RWTH Aachen University, Aachen, Germany; 2 Department of Pediatric Cardiology, University Hospital of Bonn, Bonn, Germany; 3 Institute of General Mechanics, RWTH Aachen University, Aachen, Germany

**Keywords:** Congenital heart disease, Univentricular heart, Failing Fontan, 3D real-time echocardiography, Moving mesh flow simulation, Vortex formation time

## Abstract

**Purpose:**

Patients with a functionally univentricular heart represent one of the most common severe cardiac lesions with a prevalence of 3 per 10,000 live births. Hemodynamics of the singular ventricle is a major research topic in cardiology and there exists a relationship between fluid dynamical features and cardiac behavior in health and disease. The aim of the present work was to compare intraventricular flow in single right ventricle (SRV) patients and subjects with healthy left hearts (LV) through patient-specific CFD simulations.

**Methods:**

Three-dimensional real-time echocardiographic images were obtained for five SRV patients and two healthy subjects and CFD simulations with a moving mesh methodology were performed. Intraventricular vortex formation and vortex formation time (VFT) as well as the turbulent kinetic energy (TKE) and ventricular washout were evaluated.

**Results:**

The results show significantly lower values for the VFT and the TKE in SRV patients compared with healthy LV subjects. Furthermore, vortex formation does not progress to the apex in SRV patients. These findings were confirmed by a significantly lower washout in SRV patients.

**Conclusions:**

The study pinpoints the intriguing role of intraventricular flows to characterize performance of SRVs that goes beyond standard clinical metrics such as ejection fraction.

**Supplementary Information:**

The online version contains supplementary material available at 10.1007/s13239-021-00598-9.

## Introduction

The treatment of severe congenital heart defects (CHD) in newborns is an important medical challenge of our time. In Germany, the prevalence of CHD is 1.1% of live births. 12% of these patients have severe lesions that require treatment immediately after birth.^29^

Univentricular heart (UVH) is the most common severe CHD in Germany with a prevalence of 0.03% and takes a major role in recent studies. The term functional UVH is used for heart defects in which only one ventricle is functioning and the other is absent or hypoplastic. Biventricular repair of this pathology is not possible.^28, 29^

If a severe hypoplasia of the left ventricle is present in functional UVH, it is referred to as hypoplastic left heart syndrome. Consequently, only one functional right ventricle is present and is described as singular right ventricle (SRV). This anomaly must be treated within the first days after birth with palliative measures by adjusting the blood supply to the lungs. Afterwards, during the first four years of the patient's life, the so-called Fontan circulation is achieved by means of several surgical interventions. The purpose of this procedure is to divide the single cardiac circulation into two. The Fontan procedure allows the systemic circulation to be supplied by only one ventricle. In this procedure, deoxygenated blood is diverted from the superior and inferior vena cava to the lungs by means of the total cavopulmonary connection (TCPC). Thus, only oxygenated blood is pumped into the body through the one functional ventricle.^4, 15^

The Fontan procedure is a palliative strategy for patients with single-ventricle circulation. However, its inherent limitations and deficits are becoming apparent as more and more patients are present with failing Fontan physiology.^6^ Patients with Fontan failure can develop progressive functional impairment, such as gradual systolic and diastolic ventricular dysfunction.^10^

There is a need to increase the understanding of ventricular function and physiology of Fontan patients to improve therapeutic approaches in the future. It is well known that intraventricular hemodynamics play a central role for the description of ventricular function.^25, 36^

Many studies have been performed over the years to investigate fluid dynamics in the heart and especially in the left ventricle. Among these studies, *in vivo* studies with imaging techniques (ultrasound and MRI), *in vitro* experiments with particle image velocimety (PIV), as well as numerical simulations have been performed.^7, 9, 27^

Recent studies aimed to perform flow simulations using a moving mesh method or fluid-structure interaction (FSI). As flow is strongly influenced by the motion and shape of the heart, these methods are expected to provide a more realistic representation of blood flow within ventricles. In the moving mesh method, ventricular wall motion is captured patient-specifically by imaging techniques and integrated as a boundary condition in CFD flow simulations.^3, 23, 33^ A central aspect of such studies is the investigation and evaluation of vortex formation within the ventricles.^2, 11^ Furthermore, flow analyses focus on quantitative flow energetics and blood transit properties.^22, 27, 33^ For instance, the study by Loke *et al*.^21^ has shown that vorticity and energy loss are correlated to right ventricular dysfunction in Tetralogy of Fallot patients using 4D flow MRI. The same group also used CFD simulations based on cardiac MRI data to further investigate flow parameters in this patient cohort.^20^

A few recent studies have focused on the UVH and flow imaging. Rutkowski *et al*.^26^ observed differences in kinetic energy between ventricles of Fontan patients and healthy volunteers through 4D flow MRI. Wong *et al*. analysed through 4D MRI the flows and kinetic energies in moving singular ventricles.^36^ Finally, the study by Sjöberg *et al*.^31^ determined lower diastolic ventricular kinetic energies in patients with Fontan circulation with 4D flow MRI as well. A combination of echocardiography and computational fluid dynamics (CFD) simulations was used by Chen *et al*.^5^. Real-time 3D echocardiography is a commonly used method for recording ventricular motion because it allows examination of patients with less discomfort and in less time compared to other imaging methods.^3^

The pathological SRV and the healthy LV both have to transport blood through the systemic circulation. Therefore, it was assumed in Chen *et al*.^5^ that the cardiac performance for both ventricles should be in the same range. The findings of this study underline that 3D modeling based on echocardiography is suitable to represent the morphological and functional abnormalities in the SRV during the cardiac cycle. Furthermore, CFD can detect abnormal flow patterns and vortices in the SRV compared with healthy LV. A limitation of Chen *et al*. was the consideration of three characteristic time steps throughout the diastole and steady-state simulations at these time points. In our study we expand the work by integrating ventricular motion into CFD analyses of healthy subjects (LV) and SRV from Fontan patients. We hypothesize that there are differences in fluid dynamic markers between these two groups.

## Materials and Methods

### Data Acquisition

Time-discrete ventricular volumes of five patients with single right ventricle (SRV) circulation and two subjects with healthy left ventricle (LV) were acquired from transthoraxic real-time 3D-echocardiography measurements performed at the University Hospital Bonn (UKB). The ultra-sound device iE33 (Philips Medical Systems, Andover, US, transducer X5-1) was used to record the volumetric shape of the ventricle over one cardiac cycle. Between 12 and 28 frames per heartbeat were recorded. The study was approved by the local ethics institutional review committee (Registration No. 226/06 and No. 007/15) and conformed to the principles of the Declaration of Helsinki as well as German law.

The SRV group was selected from data of Linden *et al*. and represent different ages, end-diastolic volumes (EDV), and ejection fractions (EF).^19^ The LV patients were selected from Krell *et al*. and represent small (pediatric) ventricles with EDV 40–60 mL.^14^ Table 1 summarizes the patient data used in this study.

**Table 1:** Clinical data for pathological univentricular heart (SRV) and healthy heart (LV) subjects (*EDV* 3D-end diastolic volume, *bpm* beats per minute, *EF* 3D-ejection fraction).

#	Ventricle	Gender	Age (years)	EDV (mL)	bpm	EF (%)
1	SRV	m	6	26	93	45
2	SRV	m	8	28	79	52
3	SRV	m	6	55	93	51
4	SRV	f	17	97	60	45
5	SRV	m	15	100	59	39
6	LV	m	4	40	153	67
7	LV	f	8	54	98	58

The geometric data from the echocardiography was translated into a unicode character database (UDC) format using the program ImageArena (TOMTEC Imaging Systems, Unterschleißheim, Germany). For each time step a 3D model with a constant number of cells and associated cell nodes was created. Afterwards, the UCD data was translated using Matlab into an STL file format for the application of the moving mesh model.

All STL files had the same number of nodes to facilitate interpolation between the individual time steps and the integration of continuous ventricle surface movement during CFD simulations. Additional mesh smoothing was performed with Blender 2.82.

### Simulation

Meshing of the geometry was performed with the ANSYS Meshing tool from ANSYS 2019 R1 (Ansys Inc., Canonsburg, US) with unstructured tetrahedral elements supported by a "Patch Conforming Method”. The independence of the near-wall flow mesh was determined based on the area average velocities at the inlets and outlets and the maximal wall shear stress during peak diastolic flow (E-wave) and changes in the variables of < 5% between two mesh refinement steps were considered independent. A *y* + value of < 5 was taken as a measure for appropriate resolution of the boundary layer flow. Depending on the EDV, the element number ranged from 485,000 up to 3,476,000.

For the simulations the non-Newtonian blood model by Ballyk *et al*.^1^ with a density of *ρ* = 1056.4 kg/m^3^ (hematocrit level of 44%) was applied. In addition, the pressure-based solver was selected and set in coupled mode and solved with ANSYS Fluent. The flow was assumed to be turbulent and the *k*–*ω*–SST model was applied. For the convergence of the velocity, *k*- and *ω*-residuals the criteria were set to 0.001 and for the continuity to 0.0003. The maximum number of iterations was 30. Resulting time steps ranged between 0.0018 and 0.004 s. The simulations started in the end systolic state of the cardiac cycle and ran for five cycles. The cycle independence was obtained by looking at the averaged and maximum velocity at the inlet and outlet and independence was identified after the second cycle.

As inlet and outlet conditions, patient specific pressure curves were set as boundary conditions for the SRV patients. These curves were taken from clinical data of UKB (see Linden *et al*.^19^). The pressure curves for the healthy LV have been calculated *via* a lumped parameter model (LPM) of the UKB.^35^ Valves were not modeled and a wall or a fully open inlet/outlet was set as the zone function.

The representation of a continuous ventricular movement was achieved by a moving mesh model. In brief, the model used the segmented STL geometries of each ventricle (12–28 per timestep) and, after temporal and spatial interpolation of these geometries, ventricular wall movement was implemented through the mesh motion capabilities of ANSYS Fluent by using user defined functions. A detailed explanation of the workflow with the according scripts is included in the Supplementary Material.

### Flow analysis

For the evaluation of the blood flow characteristics, vortex structure formation, vortex formation time, turbulent kinetic energies and ventricular washout were investigated.

Vortex structure formation was visualized by the Q-criterion within the ventricle.

The vortex formation time (VFT) was calculated as the time integral of the velocity from the start of the diastole to the peak of the E-wave. VFT is a dimensionless parameter describing the process of vortex formation during the beginning of diastole. It is a characteristic value for ventricular filling and it has been shown that reduced VFT correlates with deteriorated diastolic ventricular function.^12, 13^

The turbulent kinetic energy [TKE (J/kg)] is a direction-independent measure of the intensity of turbulence and represents the kinetic energy of the fluctuating velocity field.^8, 13^ The TKE was calculated in ANSYS Fluent based on the transport equation for the *k*–*ω*-SST turbulence model.^34^

The value of TKE within the ventricle was calculated for each subject as an integral value after median averaging of the last four cycles. In addition, the ratio of systolic/ diastolic TKE to total TKE was examined.

Finally, the efficiency of blood exchange within the ventricle was evaluated using washout simulations, as described in.^32^ These simulations were performed with two fluids (fluid1 = *old_blood*, fluid2 = *new_blood*) with identical rheological properties (see above).

The entire domain was initialized with *old_blood* and *new_blood* with a volume fraction of 1 at the inlet was set throughout the simulations. The flow simulation with *new_blood* was performed for three cycles gradually replacing the *old_blood*. The Volume of Fluid methodology was used to describe the interaction between the two fluids, see^32^ for further details. Results were investigated after cycle independency has been reached.

The results of the fluid analysis for the SRV group are shown as mean values and standard deviation. Furthermore, statistical comparison between the SRV group and the individual LV was performed with a one-sample *t*-test and a significance threshold of *p* = 0.01. The simulations were performed on a 64GB RAM system using 10 physical cores. Depending on the volume of the ventricles, the simulation duration was between 40 and 360 core h.

## Results

### Intraventricular flow field

**Figure 1:**
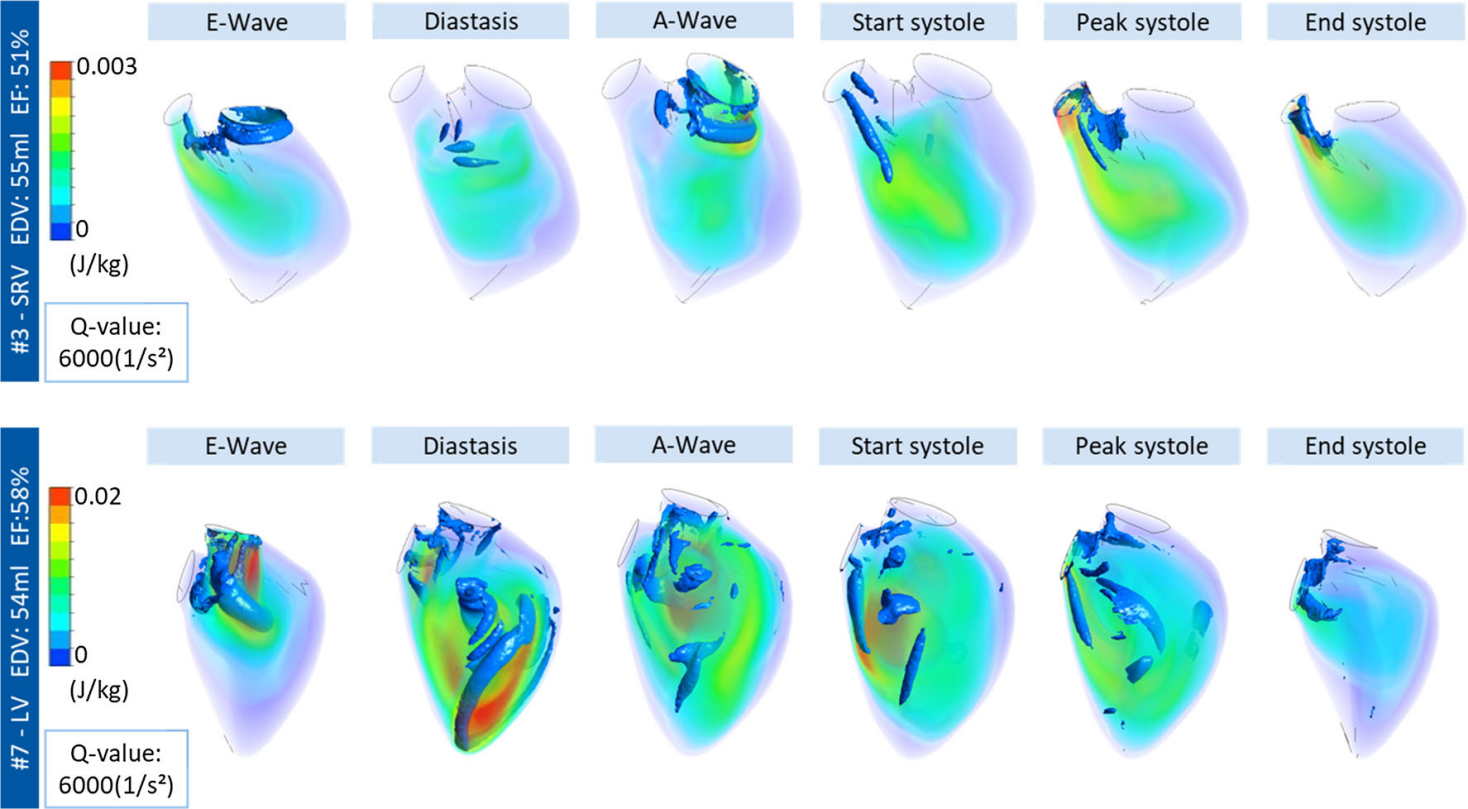
Representative turbulent kinetic energy (J/kg) and vortex structure formation with *Q*-value (1/s) for subject #3 (SRV) and subject #7 (LV). TKE represented by different scales, subject #3 max 0.003 J/kg, subject #8 max 0.02 J/kg.

Figure 1 shows the flow field inside the ventricle for subject (#3) with pathological SRV and subject (#7) with a healthy LV during diastole and systole. Six time points of the cardiac cycle are represented: E-wave, diastase, A-wave, start systole, peak systole, end systole. The blood flow over a cycle and the movement of the vortex is shown through blue iso-surfaces based on the Q-value. Furthermore, the TKE is represented in the observed time points. For better visualization of the flow within the ventricle, different scales for TKE were applied.

The presented SRV of subject #3 has an EDV of 55 mL and an EF of 51%. The beginning of the E- and A-wave can be recognized by a developing vortex at the tricuspid valve (inlet). During diastasis, the annular vortex formed at the E-wave remains only in the basal part of the ventricle. During the A-wave, another circular vortex is formed, which spreads further down than that of the E-wave, but also remains only in the basal part. Throughout systole, the vortex ring of the A-wave develops into an elongated vortex that is ejected at the end. The presented SRV-case is exemplary for the examined SRV-patients. In all cases, the vortex formation during the diastasis does not extend to the apex of the ventricle.

Exemplary blood flow in a healthy ventricle is also shown in Fig. 1. The healthy LV subject is similar in terms of volumetric conditions to the above described SRV patient. It has an EDV of 54 mL and an EF of 58%. During the E-wave, a circular vortex is formed starting from the mitral valve. During diastasis, the vortex and the TKE maximum move towards the apex. During the A-wave, the large vortices are mixed and small scattered vortices can be observed. At the beginning of systole, elongated vortices migrate to the outlet and are ejected until the end of systole.

Of note, TKE is almost an order of magnitude higher in the healthy LV subject #7 in comparison to SRV subject #3.

**Figure 2:**
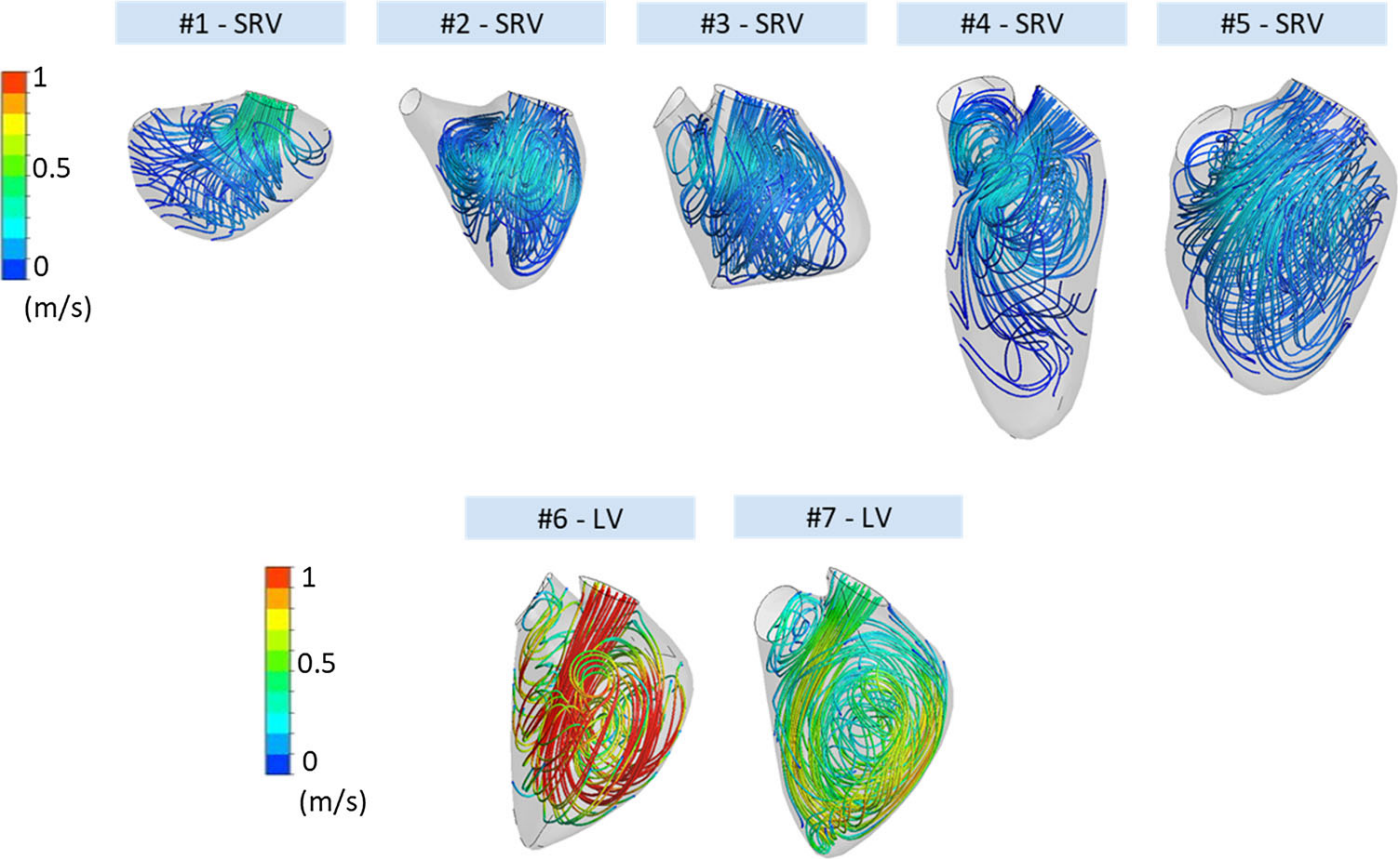
Velocity streamlines during diastasis for the SRV and LV cohorts.

Figure 2 further shows velocity streamlines during diastasis for all ventricles. The velocities in the SRV cohort are much lower than in the LV cohort with volume average velocities between 0.09 and 0.12 m/s and 0.42–0.78 m/s, respectively. As already described in the upper section, the intraventricular vortices do not progress until the apex for the SRV subjects. Of note is the strong variation of ventricular shapes in the SRV cohort.

### Fluid Dynamic Markers

The results for the flow analysis for five cardiac cycles of each patient are summarized in Table 2. The total TKE and the VFT are significantly higher for the healthy LV subjects when compared to the SRV cohort. In addition, the washout after the second and the third cycle exhibits significantly higher values for the healthy LV subjects.

**Table 2:** Results for single right ventricle (SRV) group (mean value, standard deviation) and left ventricle (LV) patient.

Flow dynamic markers	SRV	LV #6	*p*-value	LV #7	*p*-value
TKE_tot (J/kg)	0.881 ± 0.71	23.71	< 1e−5	5.44	0.0001
TKE_sys/ TKE_tot (%)	54.34 ± 16.52	44.24	0.2431	38.58	0.0998
TKE_dia/ TKE_tot (%)	45.66 ± 16.52	55.76	0.2431	61.42	0.0998
VFT (−)	1.16 ± 0.53	4.26	0.0002	3.96	0.0003
EF (%)	46.4 ± 4.71	58	0.0053	67	0.0006
Washout (%)					
1. Cycle	43.79 ± 9.77	63.05	0.0116	57.43	0.0354
2. Cycle	64.86 ± 9.54	87.26	0.0063	85.54	0.0084
3. Cycle	76.32 ± 6.64	95.51	0.0030	94.88	0.0034

*TKE* turbulent kinetic energy, *VFT* vortex formation time, *EF* ejection fraction

### Washout

**Figure 3:**
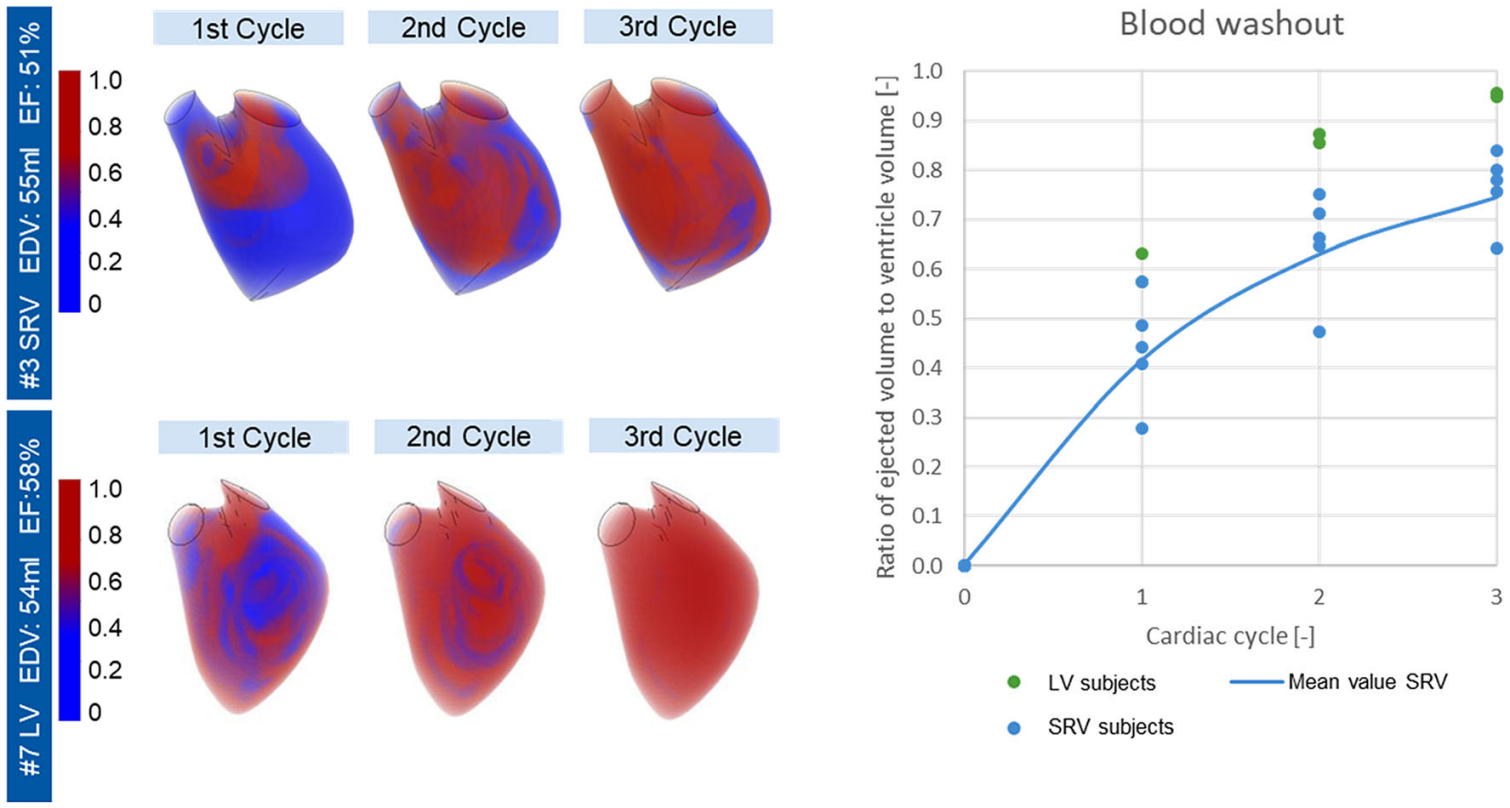
Left: Washout simulation for subject #3 (SRV) and subject #7 (LV). Time point of diastase for three cardiac cycles. Blue old blood, red new blood. Right: Volume percentage distribution of ejected volume to the total ventricle volume for SRV subjects (blue) and LV subjects (green).

The washout characteristics of SRV (subject #3) and healthy LV (subject #7) are shown in Fig. 3, left side. For three cardiac cycles the time point of diastasis is displayed. Injection of *new_blood* (red) into the SRV during the first cycle reaches only the basal part of the ventricle. The repeated injection of *new_blood* during the second cycle leads to progression of the fluid towards the apex. However, at the end of the third cycle, residual *old_blood* (blue) is still visible in the apical part of the ventricle. The SRV of patient #3 contains 74% of *new_blood* after three cycles. A lack of fluid exchange in the apical part is observed in all simulated SRV patients. In contrast, the injection of *new_blood* during diastole of the healthy LV (#7) in the first cycle already reaches the apical part. After the third cycle the ventricle is 95% filled with *new_blood*.

In the graph on the right side of Fig. 3, washout is represented by the ratio of ejected fluid to total ventricular volume for SRV (blue) and LV (green) subjects. The graph illustrates the lower washout for all SRV compared to LV. Because of the wide variation in values, a mean value curve is plotted for SRV subjects. This mean value curve shows a 20% lower value of washed out blood after three cycles compared to healthy subjects. Table 2 confirms a significant difference between the SRV patient group and the LV subjects with a threshold of *p* < 0.01 for washout after three cycles.

## Discussion

In this study, the blood flow inside pathological SRVs and healthy LVs was analyzed using real-time 3D echocadiographic data. For this purpose, data from five Fontan patients and two healthy subjects were implemented in a moving mesh simulation model. The results obtained from numerical analysis of the intraventricular flow showed significant differences in VFT, TKE_tot, and washout between SRV and LV.

### Intraventricular flow

The investigation of blood flow within the ventricle was based on the moving mesh model, which includes the movement of the ventricular inner wall as a boundary condition. The model was used to perform patient-specific analyses of cardiac flows and to investigate the differences between SRV and LV for seven subjects.

The vortex formation in healthy LV compared to pathological SRV, showed a more distinct development in healthy subjects. According to the literature, a main vortex forms in the middle of the ventricle up to the apex.^22^ With the SRV, one vortex forms at the beginning of each of the E and A waves at the inlet, splits into several elongated vertebrae, and remains in the middle of the ventricle until the end of systole. Observation of vortex formation using the Q-criterion shows that blood is not completely drawn to the apical region during diastole, which was also observed in Lampropoulos *et al*.^17^ The amount of vortex formation was qualitatively lower in the SRV compared to the LV.

EF is one of the basic evaluation factors in echocardiography for cardiac function.^24^ In the normal left ventricle, a value above 50% is taken as an indication of a patient considered healthy.^16, 18^ In Fontan patients where the SRV takes over the function of the systemic ventricle, the mean EF was 46.4%.

VFT is a dimensionless factor that has been used in several studies as an indication of an optimally forming vortex during diastole. Healthy subjects have a VFT around 4.^22^ The examined SRV subjects had VFT values of 1.16 ± 0.53, indicating a weak diastolic vortex formation and filling phase. The diastolic vortex stores kinetic energy, which initiates the ejection of blood during late diastasis. The formation of a weak diastolic vortex was also evident from the results of TKE. SRV subjects have significantly lower total TKE than the compared subjects with healthy LV. Furthermore, the ratio of diastolic TKE to total TKE is 45.7% in SRV subjects and 56–61% in LV subjects. Optimal filling and formation of TKE during diastole is necessary for sufficient energy for ventricular ejection during systole.^17^

The washout simulations illustrate the weak forming vortex during diastole and the associated inadequate filling of the entire ventricle. It was observed that residual blood remains longer in the apex of the SRV compared to the LV. After three cycles, SRV subjects have an average residual volume of 24% in the ventricle. Healthy subjects with LV have a residual volume between 5 and 7% after three cycles, similar to.^22^ The remaining blood in the apex could indicate that although the required stroke volume is provided by the ventricle, the overall fluid exchange and therefore the transport of oxygenated blood is reduced. The washout simulations in this study deliver an additional method for representing the performance of a ventricle.

During the study, a relationship between TKE and VFT that deviated from the mean was observed in two SRV subjects (#1, #3). VFT, which serves as a parameter for vortex formation during diastole, was below the mean of 1.16 (all SRV) for the two subjects (#1 = 0.62; #3 = 0.62). This indicates extremely weak diastolic filling of the ventricle. In contrast, these two subjects showed an increased total TKE (#1 = 2.178 J/kg; #1 = 1.041 J/kg) compared with the mean of 0.881 J/kg of all SRV, indicating comparatively improved overall ventricular performance. Additionally, an increased ratio of systolic TKE to total TKE (75.2%, 73.4%) was observed compared with the mean ratio of 54.3%. Overall, the results in these two patients show impaired diastolic filling and increased systolic ejection, which indicates a diastolic dysfunction of the ventricle and suggest a compensation of this dysfunction during the systole. These findings should be verified in the future by a larger subject number.

### Moving Mesh Methodology and Handling of the Geometrical Data

The advantage of the acquisition technique with the iE33 Philips ultrasound scanner is the possibility of patient-friendly and time-discrete imaging of a cardiac cycle. Real-time 3D echocardiography is a non-invasive and reliable technique that can be used at the bedside. Disadvantages of this imaging technique (related to its application in CFD simulations with a moving mesh methodology) are on the one hand the quality of the outer mesh for the geometric form. During ventricular contraction, the triangular mesh, which is constant for all time steps, causes strong deformations of the individual cells. This can lead to distorted cell nodes or illogical shapes. Manual editing of the geometry outer surface allows to obtain feasible mesh models for numerical analysis, but is time-consuming. On the other hand, the lack of proper resolution of the atrioventricular and aortic valve shows up a second disadvantage. Here additional data with e.g. transesophageal echocardiography might be an imaging modality for good resolution of the valve.

### Limitations and Outlook

Regarding this study, one of the most critical limitations was the lack of geometric information about the valvular morphology. For the data used in this study, the field of view was limited for the transthoracic ultrasound images, and the focus of the currently used software was on volumetry. The dynamics of the valve and the geometry of the atrium affect the specific properties of the inflow jet.^30^ To reduce inaccuracies in the spatial description of the motion due to the low time resolution of the data, the position parameters were primarily integrated over the total ventricular volume and cardiac cycles.

Another challenge in the evaluation of univenticular hearts is the inhomogeneous Fontan patient population. The complexity of the pathology and the surgical history of these patients is often highly individualized. Because of this limitation, we have focused only on the anatomy of singular right ventricles in this study for the time being.

Despite the small number of analyzed patients, the results already show significant differences for washout, TKE and VFT.

Further work should focus on evaluating and comparing a higher number of subjects with univentricular hearts and healthy hearts including additional information about valvular structure. The complex patient history of Fontan patients and the related anatomical conditions must always be kept in mind. To improve the usability of the acquired real-time 3D echocardiography images, methods for better mesh processing should be elaborated. Furthermore, it is important to integrate a directed flow into the simulation model, preferably under integration of heart valves. Subsequently, the simulation models require validation. This could be done in the form of a suitable PIV study or *in vivo* flow imaging such as 4D MRI.

## Conclusion

This study has shown significant differences in intraventricular flow between healthy subjects and Fontan subjects using real-time 3D echocardiography based CFD simulations. Weak vortex formation during diastole was characteristic for the examined SRV subjects. This observation was also evident in the significantly lower TKE and in the weaker washout. Numerical flow analysis presented additional parameters for the interpretation of cardiac performance besides the clinically obtained value of EF.

The studied SRV subjects had an average EF of 46%. Considering the SRV as a systemic ventricle analogous to the healthy LV, this EF suggests a mildly abnormal performance. However, the numerical flow analyses of VFT, TKE, and washout demonstrated strongly deteriorated cardiac performance. While EF is a pure volume measure, numerical analysis provided additional information on blood flow within the ventricle. By expanding the number of analyzed ventricles, these relations should be further investigated.

## Supplementary Information

Below is the link to the electronic supplementary material.

Supplementary file1 (DOCX 149 KB)
