# Supplementary material for: Intraventricular Flow Simulations in Singular Right Ventricles Reveal Deteriorated Washout and Low Vortex Formation
Source: Cardiovasc Eng Technol. 2021 Nov 30;13(3):495–503. doi: 10.1007/s13239-021-00598-9 (PMC9197806; doi:10.1007/s13239-021-00598-9)
Supplement: Supplementary file 1 — Supplementary file1 (DOCX 149 KB) [file 13239_2021_598_MOESM1_ESM.docx]

**Intraventricular flow simulations in singular right ventricles reveal deteriorated washout and low vortex formation**

**Supplementary Information**

Anna Grünwald^1^, Jana Korte^1^, Nadja Wilmanns^3^, Christian Winkler^2^, Katharina Linden^2^, Ulrike Herberg^2^, Sascha Groß-Hardt^1^, Ulrich Steinseifer^1^, Michael Neidlin^1^

**Departmental and institutional affiliations**

1. Department of Cardiovascular Engineering, Institute of Applied Medical Engineering, Helmholtz Institute Aachen, RWTH Aachen University

2. Department of Pediatric Cardiology, University Hospital of Bonn, Germany

3. Institute of General Mechanics, RWTH Aachen, Germany

**Corresponding Author**

Michael Neidlin

Email: neidlin@ame.rwth-aachen.de

**Supplementary Methods:**

***Further explanation of moving mesh method:***

The representation of a continuous ventricular movement was achieved by a moving mesh model. A schematic description of the simulation process can be seen in the Figure 1.


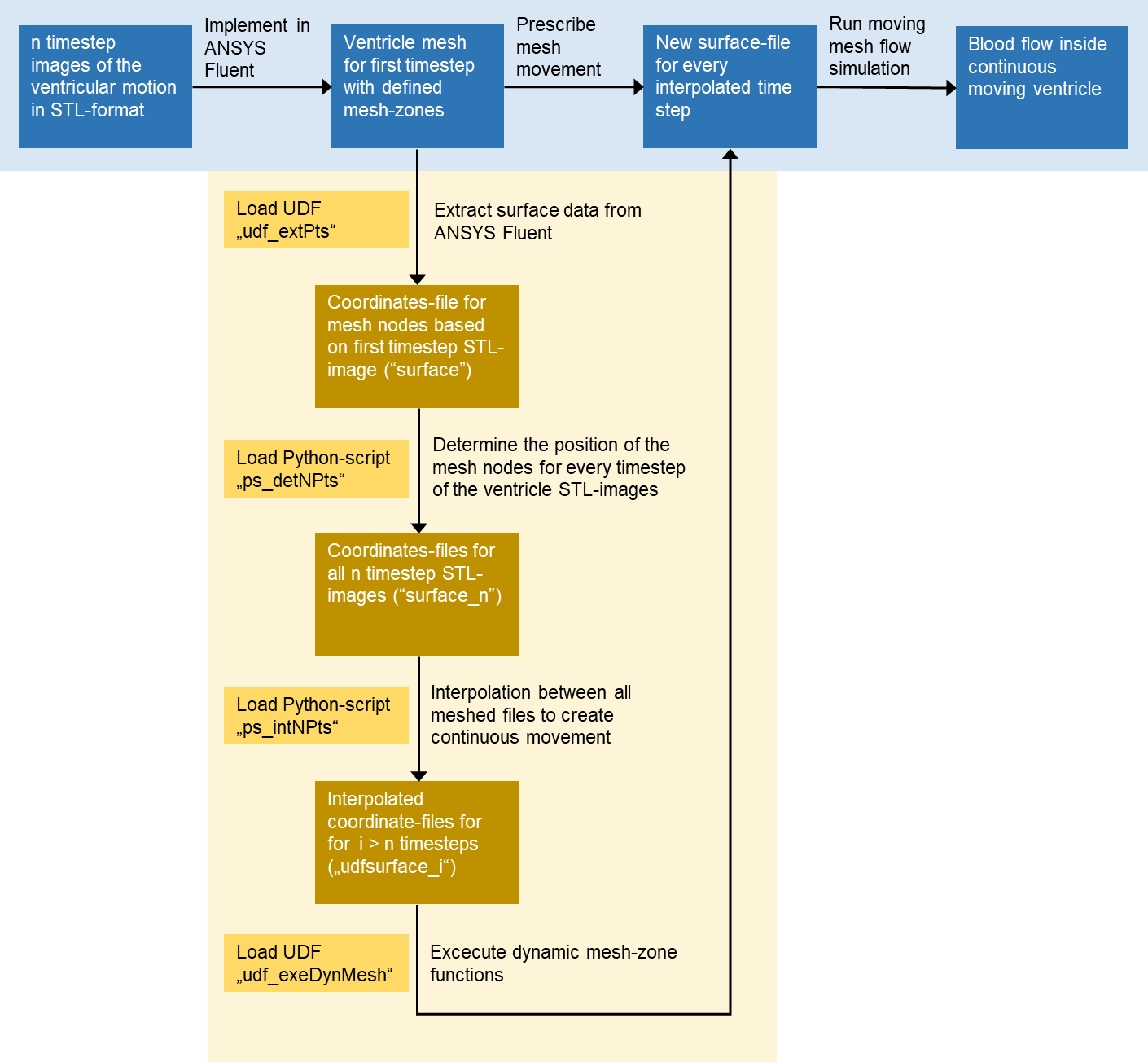


Figure 1: Implementation of ventricular movement using a moving mesh method.

For each subject, a recording of the ventricular motion with n (13-27) timesteps was provided in the STL format. The first timestep of the ventricular geometry recording was imported and meshed in ANSYS DesignModeler. The required boundary conditions and mesh zones (wall, inlet and outlet) were then implemented in ANSYS Fluent for this mesh.

In order to apply the continuous motion, the mesh points and thus the surface information were read out from ANSYS Fluent. This was done using the UDF ("udf_extPts"), which wrote the corresponding point identification number and x,y,z-coordinates for each cell node into a file ("surface"). A script ("ps_detNPts"), created with Python, was used to link the cell nodes from the “surface” file to the mesh nodes of all STL images from the ventricular recording. For each cell node from the respective timestep of the recorded STL-images, the position parameters together with the identification number of the point and the corresponding x,y,z-coordinates of the node were calculated and stored in the "surface_n" file.

Subsequently, to enable continuous ventricular motion in the simulation, temporal interpolation was performed between the mesh data. For this purpose, interpolation functions for motion were calculated for each cell node using the Python script "ps_intNPts". The regularly interpolated points were calculated between each cell node and written to the "udfsurface_i" files. Cubic spline interpolation was used as the interpolation method. The interpolation steps corresponded to the time step size set in the simulation settings (see above).

In order to load the interpolated mesh motion into the simulation, at each following interpolated timestep the corresponding surface mesh was loaded using the grid motion UDF ("udf_exeDynMesh").

The mesh model utilized smoothing and layering to ensure appropriate mesh quality during the deformation of the domain. The function “diffusion cell volume based” was used as the smoothing approach to adapt the inner mesh of the geometry to the movement of the outer surface. The diffusion parameter was set to 0, which resulted in a uniform diffusion of mesh deformation. The layering was ratio-based and dynamic, so that the inflation layers also adapted to the movement of the outer surface.

The scripts and the stl-files of SRV subject #3 are freely available on <http://www.github.com/mneidlin/movingmesh>.
